# Comprehensive Evaluation of Androgenetic Alopecia: Demographic Characteristics, Psychosocial Impact, and the Role of Social Media in Treatment Choices

**DOI:** 10.1111/jocd.70167

**Published:** 2025-04-25

**Authors:** Arzu Ferhatosmanoğlu, Zeynep Karaca Ural, Leyla Baykal Selçuk, İbrahim Etem Arıca, Deniz Aksu Arıca

**Affiliations:** ^1^ Department of Dermatology and Venerology Karadeniz Technical University School of Medicine Trabzon Turkey; ^2^ Department of Dermatology and Venerology Ataturk University Faculty of Medicine Erzurum Turkey

**Keywords:** androgenetic alopecia, female pattern baldness, quality of life

## Abstract

**Objective:**

Androgenetic alopecia (AGA) has a significant psychosocial impact on both genders. This study investigated AGA severity, psychosocial burden, sociodemographic factors, and the influence of social media on treatment‐seeking behavior.

**Methods:**

A total of 390 patients diagnosed with AGA at a university hospital in Turkey between January 2023 and 2024 were included. Data on disease severity, psychological impact, treatment awareness, and social media engagement were collected.

**Results:**

The mean AGA onset age was 23.9 years in men and 29.46 years in women. Severe AGA was observed in 38.5% of men and 41% of women, with men experiencing significantly higher emotional and functional distress (*p* = 0.05, *p* = 0.003). Early‐onset AGA (before age 20) was associated with increased psychological distress, including higher emotion, function, and stigma scores, along with lower self‐confidence (*p* = 0.007, *p* < 0.001, *p* = 0.037, *p* < 0.001). Social media was used by 69.3% of participants for hair loss information, mainly on Google, Instagram, and TikTok, but had no significant impact on treatment choices (*p* = 0.971) or psychological distress (*p* > 0.05).

**Conclusion:**

AGA significantly affects psychological well‐being, particularly in men with severe hair loss and individuals with early‐onset AGA. While social media is a primary information source, it does not influence treatment decisions or psychological distress. These findings highlight the need for evidence‐based digital health communication to counter misinformation. Future research should examine the long‐term psychological effects of AGA and further explore the role of social media in patient education and treatment behaviors.

## 1 Introduction

Androgenetic alopecia (AGA) is a common dermatological condition characterized by the progressive loss of terminal hair in a specific distribution pattern. Hair loss generally begins after puberty and is more frequently observed in males than females. The prevalence of AGA increases with age in both sexes; for white individuals, 16% of men aged 18–29 and 53% of men aged 40–49 exhibited at least moderate androgenetic alopecia (Hamilton‐Norwood III or above) [1]. Female pattern hair loss (FPHL) is most commonly observed after menopause [2]. Although AGA is considered a benign dermatological condition, its psychosocial burden can be significant. With changes in physical appearance, patients experience reduced self‐confidence, lower body image satisfaction, and decreased quality of life indices, alongside higher levels of stigmatization [3, 4, 5]. In recent years, with the increased use of the internet, the search for information and treatment options for AGA has shifted toward social media platforms. However, studies investigating this trend in AGA patients are limited.

In this study, we aim to present the disease severity and sociodemographic characteristics of male and female patients diagnosed with AGA. Additionally, we aim to assess their level of knowledge regarding treatment options, the psychological impact of the disease, whether they use social media to seek information about AGA and its treatments, and any gender‐based differences in these aspects.

## 2 Materials and Methods

Patients presenting to our department with complaints of hair loss between January 1, 2023 and January 1, 2024, who were clinically diagnosed with AGA and provided informed consent, were included in the study. The study was conducted in a university hospital in Turkey. Ethics committee approval for the study dated March 28, 2024 with protocol number 2024/2026 was obtained from the Scientific Research Ethics Committee of our Medical Faculty. Patients diagnosed with cicatricial alopecia and those under the age of 15 were excluded. Sociodemographic data, such as age, gender, height, and weight were recorded, along with details about the age of onset, duration, and severity of AGA. The severity of AGA was assessed using the Hamilton‐Norwood Scale for men and the Sinclair Scale for women. AGA Stage IVA, V, Va, VI, VII for the Hamilton‐Norwood Scale in men and FPHL Stages 3, 4, and 5 for the Sinclair Scale in women are classified as severe AGA. Patients were asked whether they used social media platforms such as Google, Instagram, Facebook, X (formerly Twitter), and TikTok to obtain information about hair loss.

The treatments used for AGA, including minoxidil spray, Platelet rich plasma (PRP), mesotherapy, microneedling, and hair transplantation, were evaluated. Additionally, popular practices among the public, such as garlic application and the use of blue water, were also examined. In our country, products marketed under names such as “blue lotion,” “blue serum,” “blue hair serum,” and “blue water” are liquid formulations with unknown contents, sold online without approval from the Ministry of Health, and claimed to promote hair growth. In this study, mesotherapy refers to hair loss prevention mesotherapy, which involves the use of vitamins, minerals, amino acids, and growth factors that nourish hair follicles.

All participants completed the Hairdex‐48 index, which consists of 48 questions designed to assess the impact of hair disorders on a patient's quality of life [6]. For each question, responses are scored on a scale from 0 (minimum) to 4 (maximum), with total scores ranging from 0 to 192, based on a 5‐point Likert scale. The index evaluates four subcategories: symptoms, function, emotion, and stigmatization. Higher scores in the symptoms, function, emotion, and stigmatization subcategories, along with lower scores in self‐confidence, indicate a lower quality of life.

In addition, participants' social media usage habits and their awareness of treatment methods for AGA were documented.

### 2.1 Statistical Analysis

The spss 23.0 statistical package program was used in the analysis of the data. The descriptive statistics of the evaluation results were given as numbers and percentages for categorical variables, and as mean, standard deviation (SD), minimum (min), and maximum (max) for metric variables. The conformity of the measurement data to the normal distribution was examined with the One‐Sample Kolmogorov–Smirnov test. In the comparisons of the measurement data of two independent groups, the Student's *t*‐test was used when the normal distribution condition was met; the Mann–Whitney *U*‐test was used when the normal distribution condition was not met. The chi‐squared test was used to analyze the differences between the categorical ratios.

Logistic regression analysis was applied to examine the effects on the dependent variable. Since the dependent variable in the analysis was binomial, the logistic regression model was preferred, and the effects of independent variables were evaluated with odds ratios. The significance of the model was tested with the Hosmer‐Lemeshow goodness of fit test, and the statistical significance of the variables was determined using the Wald test.

## 3 Results

Of the 390 patients diagnosed with AGA, 78 were female and 312 were male. The mean age of AGA onset was 23.9 years for men and 29.46 years for women. The sociodemographic characteristics of patients with AGA are summarized in Table 1.

**TABLE 1:** Sociodemographic characteristics of patients with androgenetic alopecia.

Characteristics	*n* (%)
Age (mean ± SD)	32.59 ± 9.53 (range 18–65)
**Gender**	
Female	78 (20%)
Male	312 (80%)
**Marital status**	
Married	163 (41.8%)
Single	227 (58.2%)
**Education level**	
Primary School Graduate	20 (5.1%)
Middle School Graduate	42 (10.8%)
High School Graduate	168 (43.1%)
University Graduate	123 (31.5%)
Master's Degree	18 (4.6%)
PhD	19 (4.9%)
**Employment status**	
Student	68 (17.4%)
Government employee	138 (35.4%)
Private sector employee	103 (26.4%)
Business owner	45 (11.5%)
Farmer	1 (0.3%)
Worker	5 (1.3%)
Unemployed	30 (7.7%)
**Income level**	
Minimum wage or below	61 (15.6%)
2× Minimum wage	74 (19%)
3× Minimum wage	125 (32.1%)
4× Minimum wage or above	130 (33.4%)
Body mass index	25.81 ± 4.27

The proportion of patients who developed AGA before the age of 20 was 78.2% in women and 71.2% in men. Obesity was observed in 20.5% of women and 12.2% of men with AGA, and a family history of AGA was present in 51.3% of women and 50.6% of men. Among women with AGA, family history for hair loss was observed in 51.3% of first‐degree relatives, 7.7% of second‐degree relatives, and 15.4% of both first‐ and second‐degree relatives. The corresponding figures for men were 50.6%, 12.8%, and 26.6%, respectively.

Severe AGA was classified as Grades IVa, V, Va, VI, and VII on the Hamilton‐Norwood scale for men, whereas severe FPHL was classified as Sinclair Grades 3, 4, and 5. The percentages of severe AGA were 41% in females (F) and 38.5% in males (M), respectively.

Social media usage rates were also high, with 93.6% of women and 89.4% of men using social media. Social media usage was substantial in both genders, with 84.6% of women and 78.5% of men reporting spending more than 1 h daily on the internet. Gender‐related differences in patients with AGA are summarized in Table 2. Patients were asked whether they searched on social media platforms for hair loss treatment and whether they used over‐the‐counter products for hair loss on the recommendation of friends or pharmacists. In addition, some questions were asked to investigate the level of knowledge of the patients about the methods used in the treatment of AGA. Among the patients, 69.3% used social media to obtain information about hair loss treatments. The top three most frequently used platforms for this purpose were Google, Instagram, and TikTok. Although the most well‐known treatments for hair loss among all participants were hair transplantation, anti‐hair loss shampoos, and vitamins, we found that the three most commonly used methods were anti‐hair loss shampoos, vitamins, and minoxidil spray. The top three treatments used without medical advice were anti‐hair loss shampoos, vitamin pills, and hair serums, with allergic reactions reported in 13 patients after using these products.

**TABLE 2:** Gender‐related differences in patients with AGA.

Characteristics	Female	Male
**BMI (*n*, %)**		
< 30	62 (79.5%)	274 (87.8%)
≥ 30 (Obese)	16 (20.5%)	38 (12.2%)
**Age at onset of AGA**		
< 20 years	61 (78.2%)	222 (71.2%)
≥ 20 years	17 (21.8%)	90 (28.8%)
**Duration of AGA (years)**	4.65 ± 5.24	8.33 ± 7.46
**AGA severity (Hamilton‐Norwood Scale)**		
Grade I		30 (9.6%)
Grade II		32 (10.3%)
Grade IIa		33 (10.6%)
Grade III		30 (9.6%)
Grade IIIv		44 (14.1%)
Grade IV		23 (7.4%)
Grade IVa		30 (9.6%)
Grade V		39 (12.5%)
Grade Va		27 (8.7%)
Grade VI		13 (4.2%)
Grade VII		11 (3.5%)
**AGA severity (Sinclair Scale)**		
Stage 1 (normal)	0	
Stage 2	46 (59%)	
Stage 3	25 (32.1%)	
Stage 4	6 (7.7%)	
Stage 5	1 (1.3%)	
**Severity of AGA (*n*, %)**		
Mild (AGA Grade I and II)		62 (19.9%)
Moderate (AGA Grade IIA, III, IIIA, III Vertex, IV)		130 (41.6%)
Severe (AGA Grade IVA, V, Va, VI, VII)		120 (38.5%)
Mild FPHL(Stage 2)	45 (%59)	
Severe FPHL (Stages 3, 4, and 5)	32 (%41)	
**Family history (*n*, %)**		
First degree	40 (51.3%)	158 (50.6%)
Second degree	6 (7.7%)	40 (12.8%)
First and second degree	12 (15.4%)	83 (26.6%)
None	20 (25.6%)	31 (9.9%)
**Doctor visits (*n*, %)**		
Family physician	29 (37.2%)	72 (23.1%)
Plastic surgeon	8 (10.3%)	15 (4.8%)
Dermatologist	41 (52.6%)	225 (72.1%)
**Number of doctor visits (mean ± SD)**	3.26 ± 3.30	3.70 ± 2.68
**Social media use (*n*, %)**	73 (93.6%)	279 (89.4%)
**Internet use duration (*n*, %)**		
< 1 h	12 (15.4%)	67 (21.5%)
1–3 h	43 (55.1%)	148 (47.4%)
4–6 h	12 (15.4%)	65 (20.8%)
≥ 7 h	11 (14.1%)	32 (10.3%)

A total of 146 patients (37.4%) believed that PRP treatment is suitable for everyone; 95 patients (24.6%) considered minoxidil spray to be safe during pregnancy and breastfeeding; 182 patients (53.3%) assumed that mesotherapy is solely a vitamin application; 176 patients (45.1%) believed that PRP, microneedling, and mesotherapy are more effective when used alone for the treatment of androgenetic alopecia (AGA); 116 patients (29.7%) were under the misconception that hair loss does not recur after hair transplantation; 156 patients (40%) incorrectly believed that male‐pattern hair loss is primarily caused by vitamin deficiency and that oral vitamins, biotin, and anti‐hair loss shampoos are essential for treatment; 115 patients (29.5%) assumed that PRP, mesotherapy, and laser treatments could be performed in beauty salons. Additionally, 89 patients (22.8%) were aware that hormonal treatments used for male‐pattern hair loss could potentially cause impotence. A detailed comparison of the use of social media for treatment seeking and the correct answers related to treatment methods is given in Table 3 [7, 8, 9, 10, 11, 12, 13, 14].

**TABLE 3:** Comparison of the rates of social media use for treatment seeking and true facts about AGA treatment by gender.

Category	Female (*n*, %)	Male (*n*, %)	*p*
Used social media for information on hair loss treatment	54 (69.2%)	217 (69.6%)	1.000
Used Google for information on hair loss treatment	52 (94.5%)	185 (81.5%)	**0.018**
Used Instagram for information on hair loss treatment	44 (80%)	155 (68.3%)	0.087
Used Facebook for information on hair loss treatment	19 (34.5%)	77 (33.9%)	0.524
Used X for information on hair loss treatment	24 (43.6%)	78 (34.4%)	0.130
Used TikTok for information on hair loss treatment	22 (40%)	88 (38.8%)	0.492
**Used nonprescription products for hair loss**			**< 0.001**
Self‐researched and used	17 (21.8%)	104 (33.3%)	
Used pharmacy recommendation	20 (25.6%)	91 (29.2%)	
Used friend recommendation	36 (46.2%)	59 (18.9%)	
**Treatment methods used (excluding doctor recommendations)**			
Blue water	7 (9%)	13 (4.2%)	0.085
Shampoo	38 (48.7%)	115 (36.9%)	0.069
Serum	22 (28.2%)	48 (15.4%)	**0.008**
Vitamin pills	19 (24.4%)	59 (18.9%)	0.282
Garlic, etc.	13 (16.7%)	37 (11.9%)	0.256
Experienced allergic reaction or health issues	2 (2.6%)	11 (3.5%)	0.672
**Correct answers to the following questions**			
PRP [7] treatment can be performed on everyone	50 (64.1%)	194 (62.2%)	0.430
Minoxidil [8] spray is safe during pregnancy and breastfeeding	58 (74.4%)	237 (76%)	0.768
Mesotherapy [9] is only a vitamin application	47 (60.3%)	161 (51.6%)	0.107
PRP [10], microneedling, and mesotherapy are more effective alone than together	36 (46.2%)	178 (57.1%)	0.055
Hair [11] transplantation does not result in further hair loss	51 (65.4%)	223 (71.5%)	0.180
Male [12] pattern hair loss is mainly caused by vitamin deficiency and vitamin supplements and biotin shampoos play a key role in treatment	41 (52.6%)	193 (61.9%)	0.086
Hormonal [13] treatments for male pattern hair loss can cause impotence	21 (26.9%)	68 (21.8%)	0.334
PRP [14], mesotherapy, and laser treatments can be performed in beauty salons	47 (60.3%)	228 (73.1%)	**0.020**

*Note:* Bold indicates significant *p*‐values (*p* < 0.05).

There was no statistically significant difference between the severity of AGA and the time spent on the internet in both women and men (*p* = 0.548, *p* = 0.608, respectively). No difference was observed between the time spent on the internet and treatment choice in patients (*p* = 0.971).

Patients were asked, “Which of the following treatments are you aware of?” and “Which of the following treatments have you had before?”. Approximately 51.3% of women were aware of minoxidil spray, 56.4% of PRP, 50% of mesotherapy, 29.5% of microneedling (dermapen/dermaroller), 3.8% of laser treatment, 38.5% of hormonal treatment, 75.6% of vitamin treatments against hair loss, 78.2% of anti‐hair loss shampoos, and 94.9% of hair transplantation. Among men, 41.3% were aware of minoxidil spray, 48.1% of PRP, 35.9% of mesotherapy, 27.9% of microneedling (dermapen/dermaroller), 4.8% of laser treatment, 31.1% of hormonal treatment, 60.9% of vitamin treatments against hair loss, 77.9% of anti‐hair loss shampoos, and 93.9% of hair transplantation.

Patients were asked which of these procedures they had previously undergone for hair loss. Of the women, 48.7% reported using minoxidil spray, 20.5% PRP, 25.6% mesotherapy, 11.5% Microneedling (dermapen/dermaroller), 14.1% hormonal treatment, 66.7% vitamin treatments against hair loss, 78.2% anti‐hair loss shampoos, and no one had laser and hair transplantation. In men, 17.3% reported using minoxidil spray, 18.9% PRP, 11.9% mesotherapy, 2.6% Microneedling (dermapen/dermaroller), 1% laser treatment, 2.6% hormonal treatment, 37.8% vitamin treatments against hair loss, 60.9% anti‐hair loss shampoos, and 4.5% hair transplantation.

When comparing awareness of treatment options by gender, women showed greater awareness of mesotherapy (*p* = 0.016) and vitamin treatments against hair loss (*p* = 0.015) than men. Additionally, women were more likely to use minoxidil spray (*p* < 0.001), mesotherapy (*p* = 0.002), microneedling (*p* = 0.001), hormonal treatments (*p* < 0.001), vitamin treatments against hair loss (*p* ≤  0.001), and anti‐hair loss shampoos (*p* = 0.004). Only the rate of hair transplantation was higher in men than in women (*p* = 0.048). When treatment choices were divided into two groups, minoxidil and other treatments, it was observed that in female patients, the choice of treatments other than minoxidil was statistically significantly 0.79 times higher compared to male patients.

The known and used treatment methods for androgenic alopecia, compared between genders, are summarized in Table S1.

Men with severe AGA had significantly higher emotion and function scores than men with mild/moderate AGA (*p* = 0.05, *p* = 0.003, respectively). Additionally, emotion, function, and stigmatization scores were notably higher in individuals whose AGA onset occurred before the age of 20, whereas self‐confidence scores were significantly lower in this group (*p* = 0.007, *p* < 0.001, *p* = 0.037, *p* < 0.001, respectively). There was no statistically significant difference between genders, marital status, education level, employment status, family history, social media usage (whether or not using social media), time spent on social media, use of social media to obtain information about hair loss treatment, treatment choice, and Hairdex mean scores and subcategories (Please refer to Table 4 for *p* values.). According to Hairdex total scores, no statistically significant difference was observed when the platforms used in social media were compared (*p* = 0.188 for Google, *p* = 0.485 for Instagram, *p* = 0.752 for Facebook, *p* = 0.609 for Twitter, *p* = 0.368 for TikTok, respectively). Mean Hairdex scores according to demographic and clinical characteristics are summarized in Table 4 [8, 9, 10, 11, 12]. Hairdex scores by gender are summarized in Table S2.

**TABLE 4:** Mean Hairdex scores by demographic and clinical characteristics.

Factor	Emotional impact *p* value	Function *p* value	Symptoms *p* value	Self‐confidence *p* value	Stigmatization *p* value	Overall *p* value
Gender	0.943	0.525	0.360	0.938	0.603	0.780
Marital status (Single)	0.604	0.263	0.233	0.407	0.774	0.162
Education level (University)	0.527	0.288	0.185	0.637	0.430	0.447
Employment status	0.507	0.512	0.708	0.248	0.930	0.157
Income groups	0.560	0.235	0.283	0.877	0.775	0.359
Family history	0.507	0.512	0.708	0.248	0.930	0.921
Age of onset < 20 years	**0.007**	**< 0.001**	0.452	**0.037**	**< 0.001**	**< 0.001**
**Severe AGA**						
Female	0.349	0.179	0.764	0.344	0.674	0.808
Male	**0.050**	**0.003**	0.523	0.479	0.568	0.602
Consultation with dermatologist	0.403	0.959	0.949	0.710	0.512	0.676
Social media use	0.568	0.270	0.264	0.197	0.248	0.485
High internet usage	0.456	0.280	0.392	0.943	0.065	0.188
Use of social media to obtain information about hair loss treatment	0.456	0.280	0.392	0.943	0.065	0.771
Treatment choice (minoxidil vs other treatments)	0.616	0.944	0.656	0.576	0.614	0.345

*Note:* Bold indicates significant *p*‐values (*p* < 0.05).

No differences were observed between income levels in terms of social media use for hair loss, dermatologist visits, or treatment choices. However, the frequency of Instagram use for hair loss information increased significantly with higher income levels (69.8% vs. 30.2%, *p* = 0.036).

The study form is presented in Table S3.

## 4 Discussion

Androgenetic alopecia is a prevalent dermatological condition characterized by progressive hair loss, with a notable impact on psychosocial well‐being. This study aimed to explore the severity of AGA, sociodemographic characteristics, and the influence of social media on treatment‐seeking behavior among patients. Our findings highlight several key insights into these aspects.

The average age of AGA onset was found to be 23.9 years in men and 29.46 years in women, while the duration of AGA was 8.33 years in men and 4.65 years in women. These findings are consistent with previous research, indicating that AGA may begin during adolescence or early adulthood in men, whereas it appears later in women [5, 15]. However, in our study, AGA onset before the age of 20 was found to be more common in women (78.2%) compared to men (71.2%). This may suggest a potential overrepresentation of early‐onset FPHL cases in our sample. This difference could be attributed to factors, such as self‐reported data, referred patient profiles, increased awareness, and the likelihood of earlier diagnosis in a specialized center.

In our study, the severity of AGA, as assessed by the Hamilton‐Norwood scale for men and the Sinclair scale for women, showed that 38.5% of men and 41% of women had severe AGA. In a study by Yang et al., 26.8% of men with AGA and 42.6% of women with FPHL were classified as having severe AGA [16]. Our results show that the frequency of severe AGA increases in men. The distribution of severity levels emphasizes the progressive nature of the disease, with both sexes experiencing advanced stages at a significant rate. In addition, emotion and function scores were significantly higher in men with severe AGA, highlighting the psychosocial impact of severe hair loss.

Family history of AGA was reported in 51.3% of women and 50.6% of men. In the literature, a family history of baldness has been documented in 48.5% of men and 45.2% of women with AGA [17]. In this study, we observed a similar family history prevalence among men and a slightly higher rate among women.

Recent studies indicate a notable increase in social media usage among younger patients with alopecia areata (AA) and androgenetic alopecia. Specifically, platforms like YouTube and TikTok have become increasingly popular among this demographic [18]. In a study focused on alopecia areata, the most frequently used social media platforms were Google, Instagram, and YouTube [19]. This shift highlights a growing trend in how patients seek information and support regarding their conditions.

A significant finding of this study is the high rate of social media use for information about hair loss treatments. Both men (89.4%) and women (93.6%) frequently use social media to seek information. However, there were notable differences in platform usage: women were more likely to use Instagram (80%) compared to men (68.3%), whereas men used X (34.4%) more frequently than women (43.6%). The increased use of social media platforms reflects a shift in how patients seek information, potentially influencing their treatment choices and expectations.

AGA has been closely linked to diminished body image satisfaction and an increased fear of social rejection [3]. Research indicates that individuals with AGA often experience significant psychological distress. For example, a study of Polish men found that 60% felt ashamed of their baldness, 81.3% experienced daily stress related to their condition, and 66.7% reported decreased self‐esteem due to hair loss [20]. These findings underscore the profound impact of AGA on mental well‐being and self‐perception [21]. In a study comparing AGA and AA, it was found that AGA patients experienced a greater impact on function, emotion, and symptoms, whereas self‐confidence was lower. There was no difference in stigmatization scores between the two groups [5].

Our study supports these observations, revealing significantly higher emotion and function scores in patients with severe AGA, particularly in men, highlighting the emotional burden associated with the condition. Furthermore, patients who developed AGA before the age of 20 reported higher emotion, function, and stigma scores, as well as greater functional impairment and lower self‐esteem, compared to those with later‐onset AGA.

The increasing use of social media among patients with AGA further complicates the psychological landscape. Social media platforms serve as both a source of information and a medium for social comparison, potentially intensifying feelings of inadequacy and stigma. Our study found that patients of both genders frequently turn to social media, particularly Instagram and YouTube, to seek information about hair loss treatments. This trend may contribute to heightened concerns about appearance and an increased desire for effective treatments. However, despite the prevalence of social media use, no statistically significant differences were observed in social media usage (whether or not using social media), time spent on social media, use of social media for obtaining information about hair loss treatments, treatment choice, or Hairdex mean scores and its subcategories (*p* > 0.05). This suggests that while social media plays a role in shaping perceptions, its direct influence on treatment decisions and psychological well‐being may be more complex than initially assumed.

The study also highlights gender differences in treatment awareness and usage. Women demonstrated greater awareness of mesotherapy and vitamin use and were more likely to utilize treatments such as minoxidil, mesotherapy, microneedling, hormonal therapies, vitamin supplements, and anti‐hair loss shampoos for hair loss. In contrast, men undergo hair transplantation more frequently than women. This discrepancy may stem from differing perceptions of treatment efficacy, with men potentially viewing transplantation as a definitive solution, whereas women may prefer noninvasive options that align with ongoing hair maintenance routines. Additionally, when treatment choices were divided into two categories—minoxidil versus other treatments—women were 0.79 times more likely than men to opt for treatments other than minoxidil. This could reflect differing perceptions of treatment efficacy, risk tolerance, or accessibility of treatment options.

The widespread use of nonmedical treatments, such as anti‐hair loss shampoos and vitamin pills—often without medical guidance—raises concerns about potential adverse effects, as evidenced by allergic reactions reported in 13 patients. These findings underscore the need for targeted educational initiatives to ensure that patients of both genders are well informed about all available treatment options.

Interestingly, no statistically significant differences were found between gender, marital status, education level, employment status, or family history of AGA in relation to psychological distress or treatment choices. Additionally, social media usage—whether or not patients engaged with social media, the amount of time spent online, and the use of social media to obtain hair loss treatment information—did not show any significant impact on Hairdex mean scores or treatment selection.

Despite the widespread use of social media as a source of hair loss information, the choice of platform (Google, Instagram, Facebook, Twitter, and TikTok) did not significantly correlate with Hairdex total scores (*p* = 0.188 for Google, *p* = 0.485 for Instagram, *p* = 0.752 for Facebook, *p* = 0.609 for Twitter, *p* = 0.368 for TikTok). This suggests that while social media plays a role in information‐seeking behavior, its influence on treatment satisfaction or psychological outcomes remains unclear.

Several limitations must be considered when interpreting these findings. First, the validity and reliability of the Hairdex‐48 index have not been established in Turkey. Additionally, the study was conducted at a single center, which may limit the generalizability of the results. Furthermore, self‐reported data on social media use and treatment practices may be subject to recall and social desirability biases. The cross‐sectional nature of the study precludes causal inferences about the relationship between social media use and treatment choices. Longitudinal studies are needed to better understand the impact of social media on treatment behaviors and outcomes.

## 5 Conclusion

This study highlights the psychological and functional impact of androgenetic alopecia, particularly in individuals with onset before age 20, who experience greater emotional distress, functional impairment, stigmatization, and reduced self‐confidence. In severe cases, men are more affected than women in terms of emotional and symptomatic burden. Notably, income levels did not influence social media usage for hair loss information, dermatology visits, or treatment choices, indicating that patients pursue recommended treatments regardless of financial status.

No significant associations were found between demographic factors (gender, marital status, education, employment, or family history of AGA) and psychological distress or treatment decisions. Social media engagement also did not impact Hairdex scores or treatment selection.

These findings underscore the importance of patient education and awareness in managing AGA. While social media is widely used for hair loss information, its role in treatment decisions remains unclear. Future research should further explore how social media influences patient behaviors and treatment outcomes.

## Author Contributions

**Arzu Ferhatosmanoğlu, Zeynep Karaca Ural, Leyla Baykal Selçuk:** performed the research. **Arzu Ferhatosmanoğlu, Zeynep Karaca Ural, Deniz Aksu Arıca:** designed the research study. **Arzu Ferhatosmanoğlu, İbrahim Etem Arıca, Deniz Aksu Arıca:** contributed essential reagents or tools. **Arzu Ferhatosmanoğlu, Leyla Baykal Selçuk:** analyzed the data. **Arzu Ferhatosmanoğlu, Zeynep Karaca Ural, Leyla Baykal Selçuk, Deniz Aksu Arıca:** wrote the paper. All authors have read and approved the final manuscript.

## Conflicts of Interest

The authors declare no conflicts of interest.

## Supporting information


**Table S1.** The known and used treatment methods for androgenic alopecia.

**Table S2.** Hairdex scores by gender.

**Table S3.** Questionnaire Form.

## Data Availability

The data that support the findings of this study are available from the corresponding author.
