# Supplementary material for: Comprehensive Evaluation of Androgenetic Alopecia: Demographic Characteristics, Psychosocial Impact, and the Role of Social Media in Treatment Choices
Source: J Cosmet Dermatol. 2025 Apr 25;24(4):e70167. doi: 10.1111/jocd.70167 (PMC12023709; doi:10.1111/jocd.70167)
Supplement: Supplementary file 1 — Table S1. The known and used treatment methods for androgenic alopecia. [file JOCD-24-e70167-s002.docx]

**Supplementary Table 1.** The known and used treatment methods for androgenic alopecia

| **Treatment methods known (n, %)** | Female (n, %) | Male (n, %) | p-value |
| --- | --- | --- | --- |
| Minoxidil spray | 40 (51.3%) | 129 (41.3%) | 0.073 |
| PRP | 44 (56.4%) | 150 (48.1%) | 0.117 |
| Mesotherapy | 39 (50%) | 112 (35.9%) | **0.016** |
| Microneedling (dermapen/dermaroller) | 23 (29.5%) | 87 (27.9%) | 0.439 |
| Laser | 3 (3.8%) | 15 (4.8%) | 0.717 |
| Hormonal treatments | 30 (38.5%) | 97 (31.1%) | 0.134 |
| Vitamins | 59 (75.6%) | 190 (60.9%) | **0.015** |
| Anti-hair loss shampoos | 61 (78.2%) | 243 (77.9%) | 0.951 |
| Hair transplantation | 74 (94.9%) | 293 (93.9%) | 0.747 |
| **Treatment methods used (n, %)** |  |  |  |
| Minoxidil spray | 38 (48.7%) | 54 (17.3%) | **<0.001** |
| PRP | 16 (20.5%) | 59 (18.9%) | 0.748 |
| Mesotherapy | 20 (25.6%) | 37 (11.9%) | **0.002** |
| Microneedling (dermapen/dermaroller) | 9 (11.5%) | 8 (2.6%) | **0.001** |
| Laser | 0 | 3 (1%) | 0.385 |
| Hormonal treatments | 11 (14.1%) | 8 (2.6%) | **<0.001** |
| Vitamins | 52 (66.7%) | 118 (37.8%) | **<0.001** |
| Anti-hair loss shampoos | 61 (78.2%) | 190 (60.9%) | **0.004** |
| Hair transplantation | 0 | 14 (4.5%) | **0.048** |
