# Supplementary material for: Comprehensive Evaluation of Androgenetic Alopecia: Demographic Characteristics, Psychosocial Impact, and the Role of Social Media in Treatment Choices
Source: J Cosmet Dermatol. 2025 Apr 25;24(4):e70167. doi: 10.1111/jocd.70167 (PMC12023709; doi:10.1111/jocd.70167)
Supplement: Supplementary file 2 — Table S2. Hairdex scores by gender. [file JOCD-24-e70167-s003.docx]

**Supplementary Table 2**. Hairdex scores by gender

| Female- AGA | Male- AGA | Total- AGA |  |
| --- | --- | --- | --- |
| Total hairdex | 65.84±15.39 | 66.05±17.22 | 66.01±16.85 |
| Emotions | 20.23±8.835 | 20.5±10.356 | 20.45±10.06 |
| Functions | 14.62±8.437 | 15.16±8.382 | 15.05±8.385 |
| Symptoms | 12.22±5.540 | 11.62±5.380 | 11.74±5.411 |
| Self-confidence | 27.15±6.73 | 26.96±7.327 | 26.99±7.204 |
| Stigmatization | 11.71±4.097 | 12.20±4.882 | 12.10±4.734 |
