# Supplementary material for: Comprehensive Evaluation of Androgenetic Alopecia: Demographic Characteristics, Psychosocial Impact, and the Role of Social Media in Treatment Choices
Source: J Cosmet Dermatol. 2025 Apr 25;24(4):e70167. doi: 10.1111/jocd.70167 (PMC12023709; doi:10.1111/jocd.70167)
Supplement: Supplementary file 3 — Table S3. Questionnaire Form. [file JOCD-24-e70167-s001.docx]

**CASE FORM**


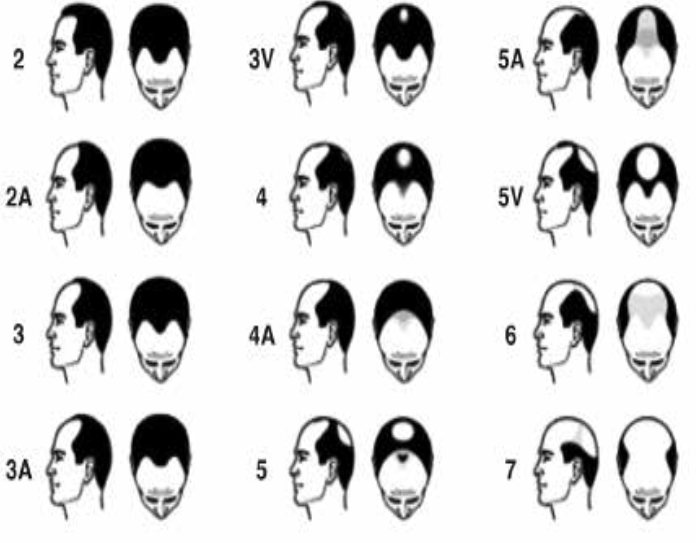


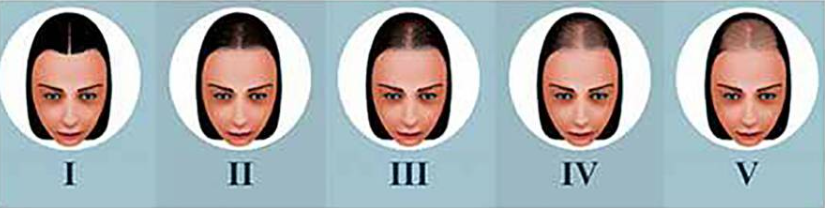


1. Age:

Weight:

Height:

1. Marital Status:
   - Married
   - Single
2. Educational Status:
   - Primary school graduate
   - Secondary school graduate
   - High school graduate
   - University graduate
   - Master’s degree
   - Doctorate
3. Employment Status:
   - Student
   - Employed in the public sector
   - Employed in the private sector
   - Self-employed (business owner)
   - Engaged in agricultural activities, including livestock care, field cultivation, and harvesting
   - Working intermittently in temporary jobs (e.g., construction, agriculture)
   - Unemployed
4. Do you have health insurance?
   - No
   - Green Card
   - Social Security Institution (SGK - ES, SSK, Bağ-Kur)
   - Other: ______
5. Total household income:
   - a. 10,000 TL or below
   - b. 10,000 - 15,000 TL
   - c. 15,000 - 20,000 TL
   - d. Above 20,000 TL
6. Do you have male-pattern hair loss (androgenetic alopecia)?
   - Yes (When did it start? ________)
   - No
7. Is there a family history of male-pattern hair loss (androgenetic alopecia) among first-degree (parents, siblings) or second-degree (uncle, aunt) relatives?
   - First-degree relatives (parents, siblings)
   - Second-degree relatives (uncle, aunt)
   - Both first and second-degree relatives
   - No
8. Have you previously consulted a doctor regarding your hair loss? How many times?
   - Consulted a general practitioner ____ times
   - Consulted a plastic surgeon ____ times
   - Consulted a dermatologist ____ times
9. Do you use social media?

- Yes
- No

1. How much time do you spend on the internet and social media daily?

- Less than 1 hour
- 1 - 3 hours
- 4 - 6 hours
- 7 hours or more

1. Have you used social media to obtain information about hair loss treatment? If yes, which platforms?

- Yes:
  - Google
  - Instagram
  - Facebook
  - Twitter
  - TikTok
- No

1. Which of the following hair loss treatment methods have you heard of?

- Minoxidil spray
- Platelet-Rich Plasma (PRP)
- Mesotherapy
- Microneedling (dermapen, dermaroller)
- Laser therapy
- Oral medications
- Vitamins (Biotin, etc.)
- Hair loss shampoos
- Hair transplantation

1. Which of the following hair loss treatment methods have you used?

- Minoxidil spray
- Platelet-Rich Plasma (PRP)
- Mesotherapy
- Microneedling (dermapen, dermaroller)
- Laser therapy
- Oral medications
- Vitamins (Biotin, etc.)
- Hair loss shampoos
- Hair transplantation

1. Have you used any hair loss products without a doctor’s recommendation?

- I researched and used them myself
- I used products recommended by a pharmacist
- I used products recommended by a friend

1. Which alternative treatments (outside of medical recommendations) have you applied for hair loss?

- Blue water solutions
- Shampoos
- Serums
- Vitamin supplements
- Garlic application
- Herbal teas
- Other: ________

1. Have you experienced any allergic reactions or adverse health effects due to these treatments?

- Yes
- No

1. Please mark the following statements as true or false:

- PRP treatment can be applied to everyone. (True / False)
- Minoxidil spray can be used during pregnancy and breastfeeding. (True / False)
- Mesotherapy is only a vitamin application (True / False)
- PRP, microneedling, and mesotherapy are more effective alone than together. (True / False)

1. Please mark the following statements as true or false:

- Hair transplantation prevents further hair loss permanently. (True / False)
- Male pattern hair loss is mainly caused by vitamin deficiency and vitamin supplements and biotin shampoos play a key role in treatment. (True / False)
- Hormonal treatments for male pattern hair loss can cause impotence (True / False)
- PRP, mesotherapy, and laser treatments can be performed in beauty salons. (True / False)

**Hair Index-48**

| **Questions** | Never | Rarely | Sometimes | Often | Always |
| --- | --- | --- | --- | --- | --- |
| 1. My scalp hurts. |  |  |  |  |  |
| 1. The condition of my hair affects my sleep pattern. |  |  |  |  |  |
| 1. I think I have a serious problem with my hair. |  |  |  |  |  |
| 4. The condition of my hair negatively affects the activities I do at work or in my free time. |  |  |  |  |  |
| 5. The condition of my hair affects my personal life. |  |  |  |  |  |
| 6. The condition of my hair makes me feel depressed. |  |  |  |  |  |
| 7. My scalp burns. |  |  |  |  |  |
| 8. Because of my hair loss, I stay at home more. |  |  |  |  |  |
| 9. I am worried that my scalp condition will result in deformation. |  |  |  |  |  |
| 10. My scalp is flaking. |  |  |  |  |  |
| 11. The condition of my hair affects my ability to get close to people I care about. |  |  |  |  |  |
| 12. I think the condition of my hair will embarrass me. |  |  |  |  |  |
| 13. I believe my hair loss will worsen. |  |  |  |  |  |
| 14. Because of my hair loss, I spend more time alone. |  |  |  |  |  |
| 15. The condition of my hair makes me angry. |  |  |  |  |  |
| 16. Water affects the condition of my hair. |  |  |  |  |  |
| 17. Because of my hair condition, I find it difficult to get close to people I like. |  |  |  |  |  |
| 18. My scalp is in bad condition. |  |  |  |  |  |
| 19. My scalp has become sensitive. |  |  |  |  |  |
| 20. The condition of my hair affects my closeness with other people. |  |  |  |  |  |
| 21. My hair loss is in a miserable state. |  |  |  |  |  |
| 22. My hair loss is a problem for my close ones. |  |  |  |  |  |
| 23. The condition of my hair makes me anxious. |  |  |  |  |  |
| 24. My hair is very sensitive. |  |  |  |  |  |
| 25. Because of my scalp condition, I spend less time with people. |  |  |  |  |  |
| 26. Because of my hair condition, I feel demoralized. |  |  |  |  |  |
| 27. My scalp is bleeding. |  |  |  |  |  |
| 28. The condition of my hair makes me aggressive. |  |  |  |  |  |
| 29. My hair loss affects my sex life. |  |  |  |  |  |
| 30. The condition of my hair irritates me. |  |  |  |  |  |
| 31. The condition of my hair is under control. |  |  |  |  |  |
| 32. The condition of my hair causes problems in places where people are close to me, such as in the cinema or on public transport. |  |  |  |  |  |
| 33. Despite the condition of my hair, I am at peace with myself. |  |  |  |  |  |
| 34. Because of the condition of my hair, I feel excluded. |  |  |  |  |  |
| 35. Despite the condition of my hair, life is beautiful. |  |  |  |  |  |
| 36. I am afraid that my hair will make me look older. |  |  |  |  |  |
| 37. The condition of my hair makes it harder for me to achieve my goals. |  |  |  |  |  |
| 38. Because of my hair condition, people make fun of me. |  |  |  |  |  |
| 39. No matter the condition of my hair, I feel confident. |  |  |  |  |  |
| 40. Other people are understanding about the condition of my hair. |  |  |  |  |  |
| 41. Despite the condition of my hair, I visit the hairdresser as usual. |  |  |  |  |  |
| 42. Because of my hair condition and hair loss, people talk behind my back. |  |  |  |  |  |
| 43. Compared to others, I am grateful for the state of my hair. |  |  |  |  |  |
| 44. The condition of my hair makes me feel unattractive. |  |  |  |  |  |
| 45. I hate seeing my hair on furniture or in my brush. |  |  |  |  |  |
| 46. I believe my hair condition is the first thing others notice about me. |  |  |  |  |  |
| 47. Every morning, I check my hair in the mirror. |  |  |  |  |  |
| 48. So far, my hair problem has not been taken seriously by doctors. |  |  |  |  |  |
